# A Prospective, Single-Center, Phase I Clinical Trial to Evaluate the Value of Transesophageal Echocardiography in the Closure of Patent Foramen Ovale With a Novel Biodegradable Occluder

**DOI:** 10.3389/fcvm.2022.849459

**Published:** 2022-05-03

**Authors:** Yajuan Du, Hang Xie, Hui Shao, Gesheng Cheng, Xingye Wang, Xumei He, Beidi Lan, Lu He, Yushun Zhang

**Affiliations:** Department of Structural Heart Disease, The First Affiliated Hospital of Xi'an Jiaotong University, Xi'an, China

**Keywords:** patent foramen ovale, interventional therapy, novel biodegradable occluder, transesophageal echocardiography, right to left shunt

## Abstract

**Objective:**

Traditional metal alloy occluders for the closure of patent foramen ovale (PFO) may be associated with some potential complications, and may restrict the trans-septal access to the left atrium for future treatment of left-sided heart disease. Increasing attention has been paid to novel biodegradable occluders (NBOs) to achieve PFO closure. We aimed to evaluate the role of transesophageal echocardiography (TEE) in the diagnostic and anatomical evaluation of PFO, as well as in the Post-procedural assessment after transcatheter closure with a NBO.

**Methods:**

We conducted a prospective, single-center clinical study of 44 patients who were diagnosed with PFO by contrast transthoracic echocardiography (c-TTE) and TEE from June 2019 to June 2020. All patients underwent PFO occlusion with NBO under TTE guidance. Follow-up was performed at 2 days and 3 months after the procedure with TTE, and at 6 months and 1 year after the procedure with c-TTE, TTE, and TEE.

**Results:**

Interventional treatment was successfully performed in all patients. The left and right sides of the occluder device disc were significantly reduced at 3, 6, and 12 months compared to 2 days after the procedure (all *P* < 0.01), and decreased gradually. The thickness was significantly reduced at 12 months compared to the first three time points (all *P* < 0.01). Thrombus was found on the surface of the occluder device in three patients (6.4%) at 3 and 6 months after occlusion. At 6 months after procedure, there were 3 (6.8%) cases of extensive residual right-to-left shunt (RLS), 2 (4.5%) cases of moderate shunt, and 7 (15.9%) cases of small shunts. One year after procedure, 2 (4.5%) cases had a extensive residual shunt, 6 (13.6%) cases of small shunts were confirmed to originate from pulmonary veins by TEE, and the PFO-RLS occlusion rate reached 95.5%.

**Conclusion:**

This study demonstrates the feasibility, safety, and effectiveness of NBO for the closure of PFO in humans, with a high rate of complete shunt closure. Accurate TEE assessment of the PFO anatomy before closure with NBO is important to ensure that the procedure remains safe and effective. Furthermore, TEE plays a crucial role in the Post-procedure follow-up.

## Introduction

Patent foramen ovale (PFO), a tunnel-like defect bounded by the primum septum and septum secundum connecting the right and left atria, is present in ~one-fourth of the general population. It has been implicated in a number of clinical syndromes, including cryptogenic stroke, migraine, decompression sickness, and platypnea-orthodeoxia syndrome (1). Transcatheter closure of PFO with occluders presents many advantages, including safety, minimal invasiveness, ease of operation, and few complications (2). However, most devices implanted in previous interventions were made of nickel-titanium alloy, which may be associated with some potential complications, and may restrict the trans-septal access to the left atrium for future treatment of left-sided heart disease (3). Beyond traditional permanent metallic devices, NobleStitch EL, a suture-based system, has been developed as an alternative approach limiting the burden of exogenous material released at the cardiac level. However, despite promising, only few data are available detailing the effectiveness and the safety of the NobleStitch system (4, 5). Biodegradable occluders effectively avoid the disadvantages of metal occluders. Thus, in the past decade, partly or totally biodegradable occluders have attracted increasing attention (6). However, many biodegradable occluders have been limited to animal experiments, and only a few devices are used in clinical experiments (7–10). No research has yet explored the medium- and long-term effects of clinical experiments using a biodegradable occluder systematically monitored by transesophageal echocardiography (TEE). TEE plays a major role in the diagnostic evaluation of PFO, as well as in Post-procedural assessment after transcatheter closure. In this clinical context, we provide a prospective, single-center, phase I clinical trial to evaluate the value of TEE in the closure of PFO with novel biodegradable occluders (NBOs).

## Materials and Methods

### Patient Population

The feasibility study was designed as a prospective, single-center, first-in-human trial. We prospectively investigated 44 PFO patients (21 men, 23 women; mean age 36.6 ± 10.9 years) after excluding those with a small ASD, who admitted to the Department of Structural Heart Disease in our hospital from June 2019 to June 2020 suffering from transient ischemic attack (TIA), cryptogenic stroke, and migraine headaches. All patients or their relatives provided written informed consent to participate in the study prior to the study. The study protocol was approved by the ethics committee of the First Affiliated Hospital of Xi'an Jiaotong University (No: XJTU1AF2015LSL-049). Routine ultrasound, computed tomography (CT), and magnetic resonance imaging (MRI) were used to rule out cardiac, intracranial, extracranial arterial disease, and pulmonary arteriovenous malformations.

### Transthoracic Echocardiography (TTE) and TEE Examination

TTE was conducted using the GE Vivid E9 platform equipped with a 1.5–4.6 MHz M5S transducer (Horten, Norway). TTE was performed to acquire the parasternal aortic short-axis view, parasternal 4-chamber view, and subcostal biatrial view. TTE was used to observe the morphology of the atrial septum, whether there was “interleaved-like change” in the primary and secondary septa. Color flow Doppler was used to observe the presence of a left-to-right shunt (LRS) at the foramen ovale of the interatrial septum. Contrast transthoracic echocardiography (c-TTE) was used to identify the RLS. The modified c-TTE procedure was performed according to the methods described in our previous study (11). RLS was graded according to the highest number of microbubbles observed in the left chamber in a single frame as follows: image-negative (no microbubbles), small (1–10 microbubbles), moderate (11–30 microbubbles), or extensive (30 microbubbles or left chamber opacification) (12).

**Figure 1:**
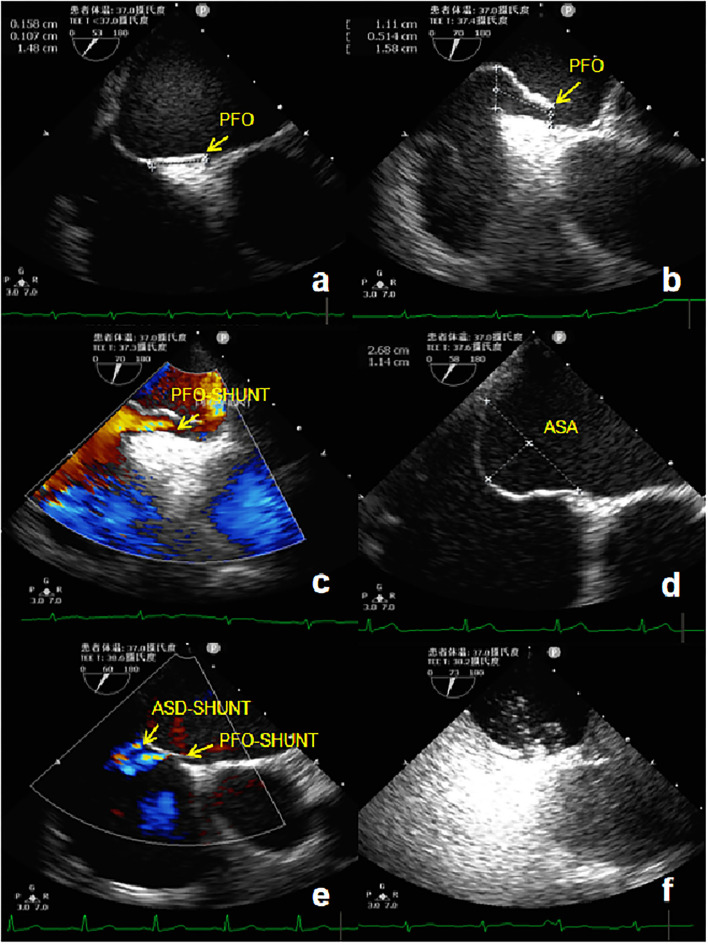
TEE detection of PFO. **(a)** Two-dimensional TEE displaying a “slit-like” channel of PFO at rest. **(b)** Two-dimensional TEE displaying a “slit-like” channel of PFO during Valsalva maneuver. **(c)** Doppler color flow imaging showing spontaneous PFO left-to-right shunt. **(d)** Two-dimensional TEE displaying an ASA. **(e)** Doppler color flow imaging showing spontaneous the left-to-right shunt of PFO and small ASD. **(f)** TEE bubble study showing microbubbles passing through the PFO. TEE, Transesophageal echocardiography; PFO, Patent foramen ovale; ASA, Atrial septal aneurysm; ASD, Atrial septal defect.

TEE was performed using the GE Vivid E9 platform fitted with a 3.0–8.0 MHz multi-frequency probe. A standardized TEE protocol was used to assess the morphologic characteristics of the atrial septum and RLS through a PFO with agitated saline, both at rest and during the Valsalva maneuver (13). A TEE 50°-70° special view at the mid-esophagus was used to measure the left side size, right side size, and the length of the PFO tunnel at rest and after the Valsalva maneuver. If the expansion range of the atrial septum was ≥6 mm, the basal width and expansion range were measured (Figure 1). TTE and TEE were all performed and observed by the same experienced sonographer.

### PFO Occluder Device and Implantation Technique

**Figure 2:**
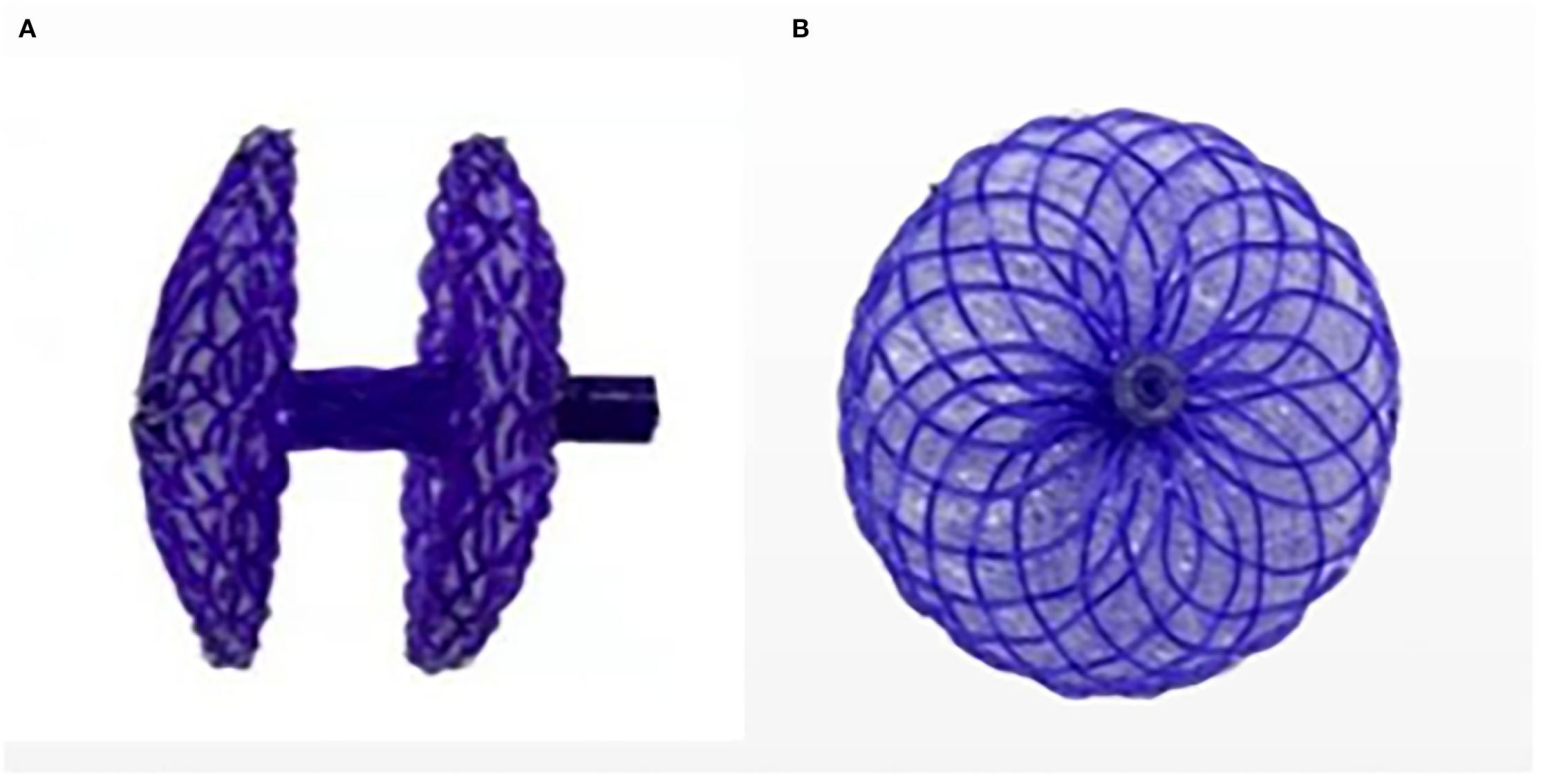
Novel biodegradable Occluder. **(A)** Side view of the occluder device. **(B)** Positive view of the occluder device.

The PFO-NBO used in this study was provided by the Shanghai China JinKui Medical Occluder Device Company. This occluder device has a double-disc single rivet shape (Figure 2). The bilateral disk and right disc riveting were made of polydioxanone (PDO) filaments. Both discs were filled with polyethylene terephthalate (PET) nonwoven fabric. The NBO structure was self-bulging. Double-sided discs, waist, and right disc riveting were made from a PDO single filament of 0.298 mm diameter. Both discs were flat, with a circular bulge of 2 mm in the center of the right disc, an outer diameter of ~3 mm, an internal threaded structure for fixing the transfer lever, and an occluder waist height of 4 mm. PDO has good shape memory ability, so it can be completely compressed and collected into the catheter for profit transmission and release. It is presently available in 7 sizes: 18/18, 24/18, 24/24, 30/24, 30/30, 30/34 and 34/34 mm for the right and left atrial disc size, respectively.

The procedures were performed with the patients under local anesthesia using digital subtraction angiography (DSA) and TTE imaging. After cannulation of the right femoral vein, a soft-tipped guidewire was advanced through the PFO and positioned within a left-sided pulmonary vein. All patients received intravenous heparin (100 IU/kg) during the procedure. After rehydration in heparinized saline, the occluder device was loaded into a proprietary delivery catheter and advanced to the left atrium via an 11 F transseptal sheath. The distal umbrella was opened in the left atrium, and the occluder device and sheath were retracted until the distal umbrella was opposed to the left atrial wall of the septum. The proximal umbrella was deployed by further withdrawal of the sheath. Correct positioning of the occluder device was confirmed by two-dimensional and color Doppler TTE imaging in multiple planes, after which the delivery system was activated to release the implant.

### Management After Implantation

**Figure 3:**
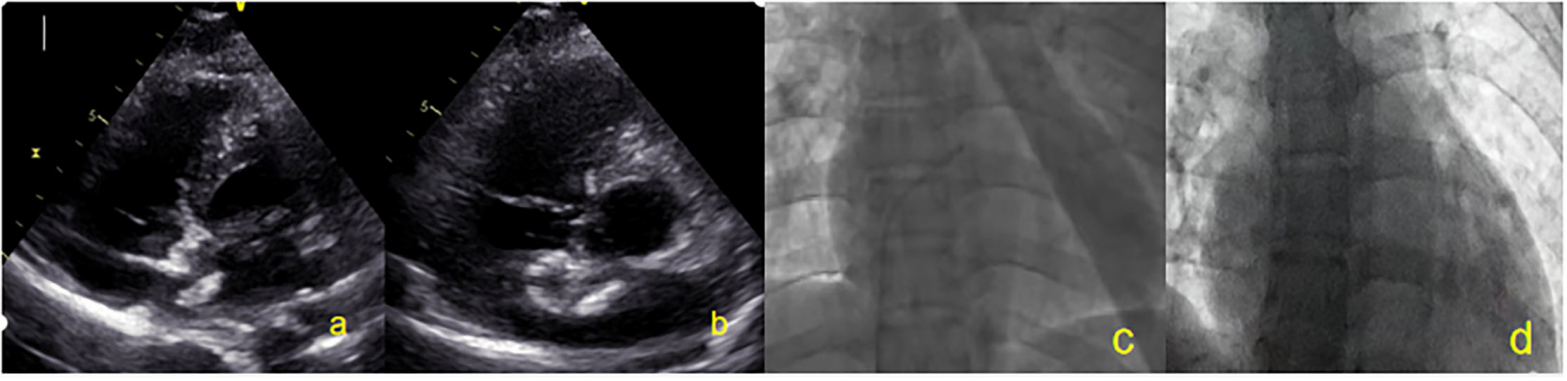
All patients were closured by TTE and DSA. **(a)** Two-dimensional TTE displaying left disc release. **(b)** Two-dimensional TTE displaying right disc release. **(c)** DSA showing the catheter entered the left atrium. **(d)** After the occluder is released, the invisible occluder is displayed by DSA. TTE, Transthoracic echocardiography; DSA, Digital subtraction angiography.

On the second day after closure, patients underwent TTE to confirm the correct occluder device position. Aspirin 100 mg/day is recommended to be used for 6 months after the procedure and clopidogrel (50 mg/day) is recommended to be used for 3 months after PFO closure. TTE and c-TTE were performed at baseline (before closure), 6 months, and 1 year after closure. Residual shunts were assessed using c-TTE. TTE was performed only at 2 days and 3 months; TEE was also repeated at 6 months and 1 year to assess the change in the occluder device size and thrombus formation. A TTE parasternal 4-chamber view and a TEE 50°−100° special view at the mid-esophagus were respectively used to measure the thickness, left side and right side diameters of the NBO (Figure 3). Echocardiography was performed by the same experienced sonographer. At each visit, an electrocardiogram was used for screening of arrhythmia, and blood was taken for measurement of hematologic and biochemical parameters, and C-reactive protein.

## Statistical Analysis

Continuous variables are expressed as mean ± standard deviation, and categorical variables are reported as counts and percentages. Unpaired *t* test was used to compare the size and thickness change of the NBO after occlusion between different periods. Statistical significance was assumed when the *P*-value was < 0.05. All data were analyzed using the SPSS software (version 18.0.1, SPSS Inc.).

## Results

### Basic Information on the Study Subjects

Forty-four patients with PFO were included in this study. Their basic information is shown in Table 1.

**Table 1:** Patient baseline demographics characteristics of the study population (*n* = 44).

**Clinical features**	***N* (%)/Mean ±SD**
Age (year) mean standard deviation	36.6 ± 10.9
Sex (male)	21 (47.7)
Sex (female)	23 (52.3)
Coronary heart disease	1 (2.3)
Hypertension	8 (18.2)
Diabetic mellitus	1 (2.3)
Arrhythmia	2 (4.5)
Indication for closure	
Cryptogenic stroke	5 (11.4)
Transient ischemic attack	3 (6.8)
Migraine	28 (63.6)
c-TTE before closure	
Positive at rest	15 (34.1)
Extensive RLS after Valsalva maneuver	44 (100)
PFO size After Valsalva maneuver by TEE	
PFO size of right atrial side (mm)	3.9 ± 0.3
PFO size of left atrial side (mm)	1.7 ± 0.1
PFO length of tunnel (mm)	7.4 ± 0.4
Other special structures by TEE	
Atrial septal aneurysm	1 (2.3)
Atrial septal soft	2 (4.5)
Long tunnel	10 (22.7)
Occluder device size (mm)	
24/18	3 (6.8)
24/24	40 (90.9)
30/30	1 (2.3)

*c-TTE, Contrast transthoracic echocardiography; TEE, Transesophageal echocardiography; PFO, Patent foramen ovale*.

### Results of Transcatheter Closure

**Figure 4:**
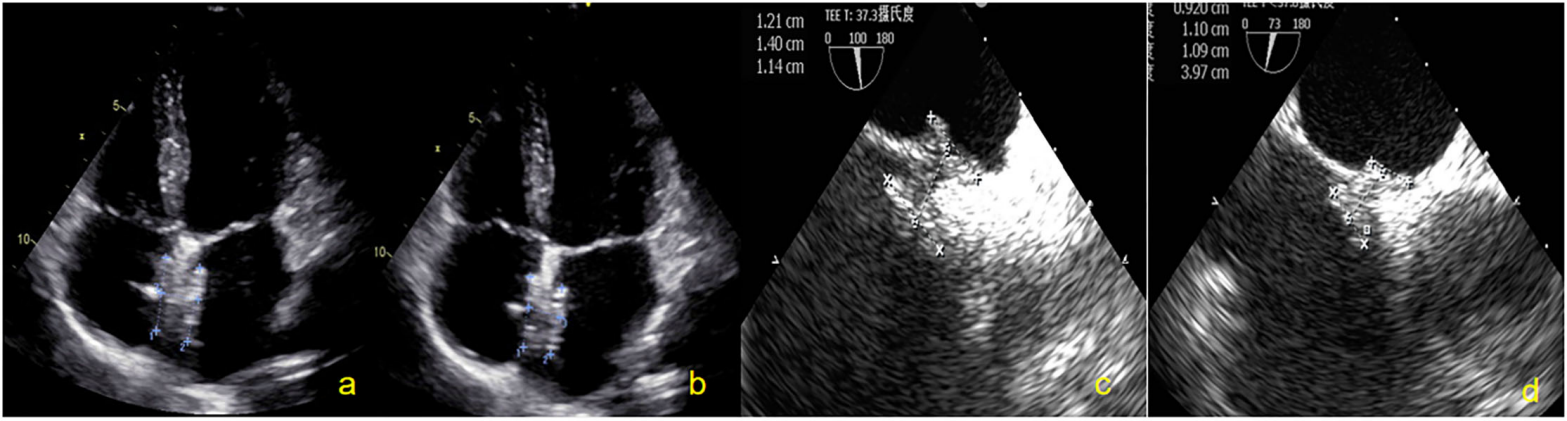
Subjects were followed up by TTE and TEE. **(a)** Two-dimensional TTE displaying the size of the NBO at 2 days. **(b)** Two-dimensional TTE displaying the size of the NBO at 3 months. **(c)** Two-dimensional TEE showing the size of the NBO at 6 months. **(d)** Two-dimensional TEE showing the size of the NBO at 12 months. NBO, Novel biodegradable occlude; TTE, Transthoracic echocardiography; TEE, Transesophageal echocardiography.

Most patients (40/44, 90.9%) were implanted with 24/24 mm occluders, 3 (6.8%) patients with smaller PFO were implanted with 24/18 mm occluders, and 1 (2.3%) patient with ASA was implanted with a 30/30 mm occluder. DSA cannot display the occluder image during the entire process of occluder release. All patients successfully underwent PFO occlusion with NBO under TTE guidance (Figure 4). The closure success rate was 100%.

### The Size and Thickness Change of NBO After Occlusion

TTE was used to measure the diameters and thickness of the occluder device at 2 days and 3 months after closure. The TTE image of the NBO was clear during this period. NBO was measured using TEE at 6 and 12 months. The results showed that the left and right diameters of the occluder device disc were significantly reduced at 3, 6, and 12 months, compared to those at 2 days (all *P* < 0.01), and decreased gradually. The left and right diameters of the occluder at 12 months were respectively only 11.5 ± 0.4 mm and 12.1 ± 0.4 mm (the original diameters: 24.2 ± 0.4 mm and 24.5 ± 0.3 mm). The thickness of the occluder device decreased slightly at 3 and 6 months, compared to those at 2 days (all *P* > 0.05), and the thickness was significantly reduced at 12 months compared to the first three time points (all *P* < 0.01). The diameters and thickness changes of the occluder plate after occlusion are shown in Table 2.

**Table 2:** Size and thickness change of occluder plate after closure.

**Size and thickness**	**2 days (TTE)**	**3 months (TTE)**	**6 months (TEE)**	**12 months (TEE)**
Left disk (mm)	24.2 ± 0.4	20.1 ± 0.3 ^a^	13.5 ± 0.4 ^ab^	11.5 ± 0.4 ^abc^
Right disk (mm)	24.5 ± 0.3	20.4 ± 0.3^a^	13.5 ± 0.3 ^ab^	12.1 ± 0.4 ^abc^
Thickness (mm)	12.2 ± 0.2	12.1 ± 0.2 ^d^	12.0 ± 0.4 ^de^	10.5 ± 0.4 ^abc^

*Compared with 2 days (TTE), a indicates P < 0.01; d indicates P > 0.05; Compared with 3 months (TTE), b indicates P < 0.01; e indicates P > 0.05; compared with 6 months (TEE), c indicates P < 0.01*.

*TTE, Transthoracic echocardiography; TEE, Transesophageal echocardiography*.

### Complications After Occlusion

**Figure 5:**
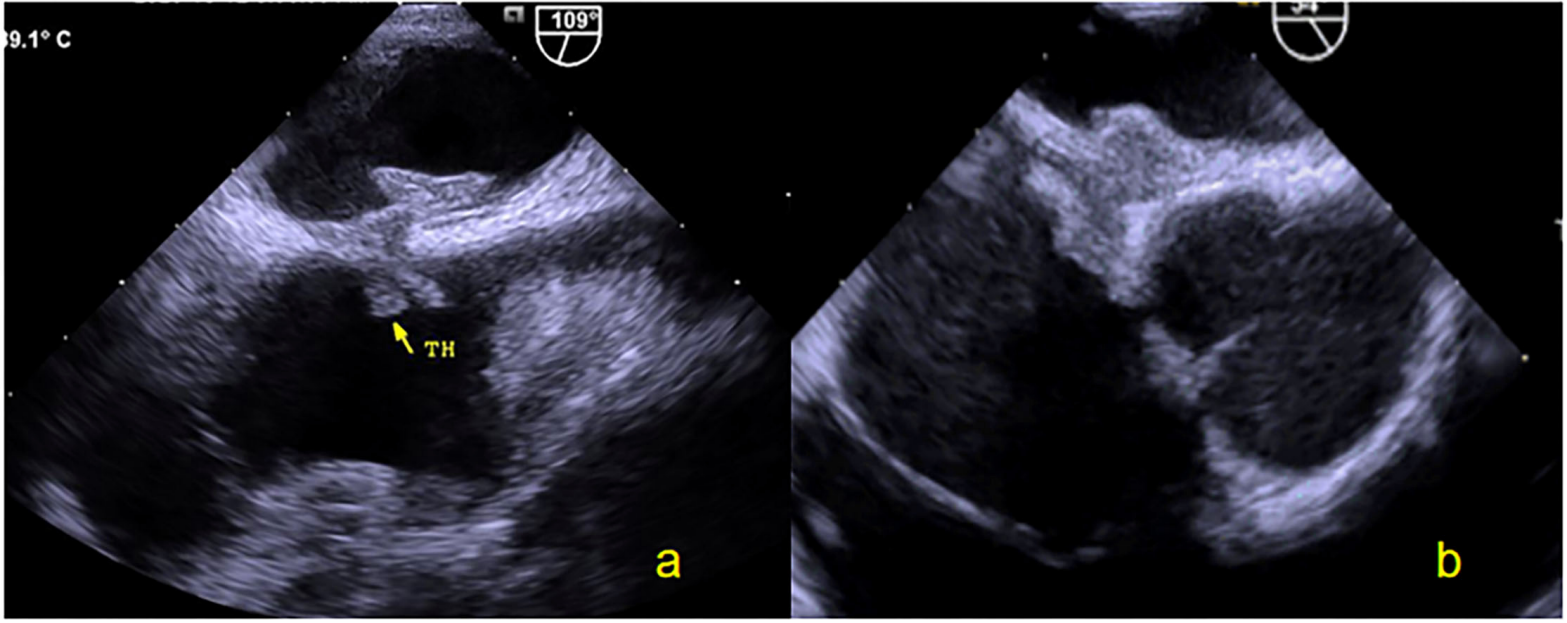
Thrombus observed by TEE. **(a)** Thrombus was found on the surface of the right occluder device by two-dimensional TEE at 6 months. **(b)** Thrombus of the right occluder device disappeared after 1 month. TEE, Transesophageal echocardiography.

All patients completed TTE at 3, 6, and 12 months and c-TTE and TEE at 6 and 12 months after occlusion. The results showed thrombi on the surface of the occluder device in 3 (6.8%) patients at 3 and 6 months after occlusion. The TEE was reviewed after 1 month of intensive anticoagulation treatment (taking rivaroxaban 20 mg/day) and all abnormal clumps disappeared (Figure 5). No clinical adverse events occurred, such as acute stroke and acute peripheral artery embolism et al. At 6 months after procedure, there were 3 (6.8%) cases of l extensive residual RLS, 2 (4.5%) cases of moderate shunt, and 7 (15.9%) cases of small shunt. One year after operation, the TEE result showed that 2 (4.5%) cases with extensive shunt all exhibited an intradisc shunt and 6 (13.6%) cases of small shunt were confirmed to originate from pulmonary veins. The PFO-RLS occlusion rate reached 95.5% (Table 3). There was no evidence of novel arrhythmia or other systemic adverse responses in any patient.

**Table 3:** Residual shunt rate after closure (*N* = 44).

**RLS grading**	**6 months**	**12 months**
Negative	32 (72.7%)	36 (81.8%)
Residual RLS	12 (27.3%)	8 (18.2%)
Mild	7 (15.9%)	6 (13.6%)
Moderate	2 (4.5%)	0 (0%)
Extensive	3 (6.8%)	2 (4.5%)

*RLS, Right-to-left shunt*.

## Discussion

Four randomized controlled trials have reported that transcatheter PFO closure plus antiplatelet treatment is superior to anti-platelet therapy alone for secondary stroke prevention (14–17). Therefore, interventional treatment of PFO has been increasingly valued by experts both at home and abroad. In 1997, Swiss scholars Amplatzer and Meier launched the first PFO closed occluder device, termed the Amplatzer occluder device. With a simple implantation process, good sealing effect, and high safety, this device has been widely used in clinical practice. However, adverse reactions of occlusion displacement, serious arrhythmia, and other conditions after interventional occlusion can occur. Additionally, nickel-titanium alloy material occluder devices remain in the body for a long time, which may be associated with some potential complications, and may restrict the trans-septal access to the left atrium for future treatment of left-sided heart disease (18–20). Beyond traditional permanent metallic devices, NobleStitch EL, a suture-based system, has been developed as an alternative approach closuring the PFO. However, despite promising, only few data are available detailing the effectiveness and the safety of the NobleStitch system (4, 5). Thus, biodegradable occluders have become research hotspots for PFO interventional therapy in recent years. TEE has a major role in the diagnostic evaluation of PFO, as well as in diagnosing potential outcomes or complications such as device embolization, device thrombosis, infective endocarditis, device erosion and cardiac or extracardiac residual RLS on Post-procedure follow-up. The goals of this study were to explore the value of TEE in the preoperative evaluation and postoperative follow-up of closed PFO with NBO.

In the present study, all cases were confirmed as PFO by TEE with agitated saline, and patients with pathological pulmonary arteriovenous fistula as well as those with a small ASD were excluded. Preoperative TEE results showed that there were 17 cases with size >2 mm (maximum, 4.5 mm), 1 case with ASA, 2 cases with high-mobility atrial interval, 10 cases with long tunnel (14 mm maximum). No attempt was made to select patients with more favorable anatomy, and no patient with a PFO was excluded on the basis of defect size or presence of an aneurysm. Many previous studies have reported the importance of TEE in the preoperative diagnosis of PFO and detailed anatomical structure assessment (16, 21, 22). The NBO production materials used in this study were PDO filament and PET nonwoven fabric; PDO is biodegradable polyester ether, and both discs were filled with nonwoven PET fabric to prevent blood flow. PDO is now widely used in biomedical and pharmaceutical applications such as surgical implants, sutures, drug carrier and tissue engineering scaffold. With nearly 50 years of use in humans since their introduction as sutures, these polymers are generally regarded as safe and have been approved by the US Food and Drug Administration (23). PDO can be decomposed into CO2 and H2O, and it has been proven to be very suitable for making the congenital heart disease-related occluder because of its biocompatibility and high safety (24). The size of the NBO was selected individually according to the TEE results. In this study, most patients (40/44, 90.9%) were implanted with 24/24 mm occluders, 3 (6.8%) patients with smaller PFO were implanted with 24/18 mm occluders, 1 (2.3%) patient with ASA was implanted with a 30/30 mm occluder. DSA cannot display the occluder image during the entire process of occluder release. All patients were successfully closed under the TTE guidance. The operation success rate was 100%, and none of the patients had complications of occluder displacement or falling off.

**Figure 6:**
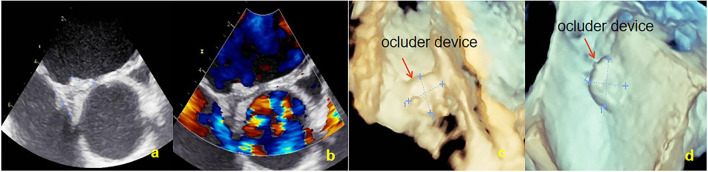
Overall outline of the device was observed by TEE at 12 months; both discs were approximately circular and completely fitted the atrial septum. **(a)** Two-dimensional TEE displaying the shape of NBO. **(b)** Doppler color flow imaging showing that there was no shunt in the atrial interval. **(c)** Three-dimensional TEE displaying the outline of the right side occluder. **(d)** Three-dimensional TEE displaying the outline of the left side occluder. TEE, Transesophageal echocardiography; NBO, Novel biodegradable occluder.

After implantation of the NBO, the tissue around the occluder device grew and gradually completed endothelialization. The role of the occluder device is to provide a temporary “bridge” for occluder device endothelialization. After completing the “bridge” role, the support structure of the occluder device can be completely absorbed and degraded into small molecules that are harmless to the human body. The results showed that the left and right sides of the occluder device disc were significantly reduced at 3, 6, and 12 months compared to that at 2 days (all *P* < 0.01), and gradually decreased. A clear occluder device profile was still observed by TEE at 12 months after occlusion, but the left and right diameters were respectively only 11.5 ± 0.4 mm and 12.1 ± 0.4 mm (the original diameters: 24.2 ± 0.4 mm and 24.5 ± 0.3 mm). A well-seated PFO occluder device was placed to cover the patent foramen ovale, and both discs were approximately circular and completely fitted the atrial septum (Figure 6). The thickness was significantly reduced at 12 months (10.5 ± 0.4 mm) compared to the first three time points (all *P* < 0.01). In our previous animal experiments, pathological examination confirmed that the skeleton began partially collapse from the edge at 3 months after occlusion, but that the structure was still intact, and the surface was coated with continuous flat-shaped cells. The skeleton was collapsed and replaced with collagen fibers, and fibroblasts and fibroblasts were seen inside at 6 months. The occluder skeleton was replaced by granulation tissue, and new capillaries and fibroblasts could be seen on the basis of fibroconnective tissue at 12 months (25). With the granulation process and fibroconnective tissue formation, some negative remodeling consequences (such as interatrial septum stiffness, tumor formation and pseudoaneurysm) may emerge. The postoperative follow-up device size changes in this study were consistent with the results of our previous animal study. The overall outline of the FBO was significantly reduced with the degradation of the occluder device. Although the local atrial septum remained thick and stiff, a sufficiently long residual atrial septum makes the puncture through the atrial interval easier for future treatment of left-sided heart disease (such as electrophysiology ablations, percutaneous mitral valve repair/implantation, left atrial appendage occlusion, paraprosthetic valve leak repair and left ventricular assist device positioning et al.) (26). At present, there are few studies on degradable occluder, no long-term follow-up results, and no reports of tumor and pseudoaneurysm formation after closure by degradable occluder.

**Figure 7:**
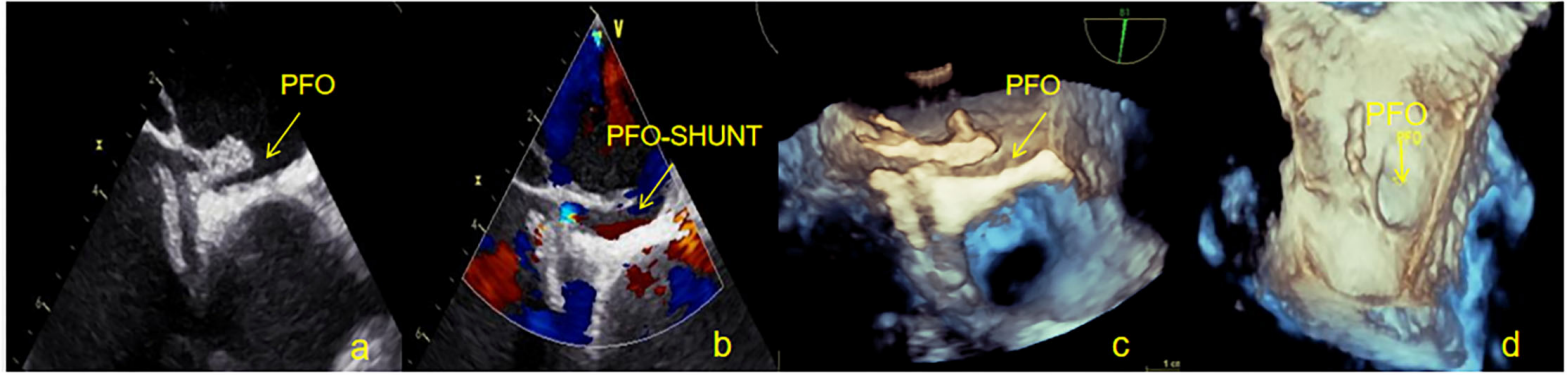
**One** patient with a large residual right to left shunt was observed by TEE at 12 months. **(a)** Two-dimensional TEE displaying the PFO gap remains open in the occluder device. **(b)** Doppler color flow imaging showing the left to right shunt of the PFO. **(c)** Three-dimensional TEE displaying the outline of the occluder. **(d)** Three-dimensional TEE displaying the outline of the left side occluder, where the left atrial side of the PFO remains open. TEE, Transesophageal echocardiography; PFO, Patent foramen ovale.

There was no evidence of novel arrhythmia or allergic reactions were identified in our study. Thrombus was found on the surface of the occluder device in 3 patients (6.4%) at 3 and 6 months after occlusion. However, no clinical adverse events occurred, such as acute stroke and acute peripheral artery embolism. The incidence of device thrombosis was reported to be 0.4–2% after PFO closure (14, 27–29). Thrombus appears to be more common with devices containing uncoated metal arms than with the polyester fabric-coated device such as the Amplatzer. Most thrombi were detected by TEE within the first month after device implantation. Patient compliance with antiplatelet therapy is required to prevent thrombosis. The incidence of thrombosis in this study was higher, 2 of 3 patients were considered due to spontaneous discontinuation of antiplatelet drugs. The TEE was reviewed after 1 month of intensive anticoagulation treatment (taking rivaroxaban 20mg/day), at which point all thrombi had disappeared. There were 3 (6.8%) cases of extensive residual RLS, 2 (4.5%) cases of moderate shunt, and 7 (15.9%) cases of small shunt at 6 months. One year after the operation, the TEE result showed that 2 (4.5%) cases with extensive shunt all exhibited an intradisc shunt and 6 (13.6%) cases of small shunt were confirmed to originate from pulmonary veins, and the PFO-RLS effective occlusion rate reached 95.5%. In our previous study, we enrolled 246 patients (105 men) with a PFO. All patients were treated by PFO interventional closure, with the Cardi-O-fix PFO occluder being used in 180 patients and the Amplatzer PFO occluder being utilized in the remaining 66 patients. The effective closure rates of the Cardi-O-fix and Amplatzer PFO occluder devices at the 12 months after the procedure were 90.6 and 86.4%, respectively (30). In many other clinical studies, residual shunt may be observed in up to 25% of patients after PFO closure, and nearly 10% show moderate to large residual shunting (15, 16, 31). Compared with these results, the effective closure rates of the NBO was higher. According to the results of TEE in our study, the causes of extensive residual PFO-RLS in 1 year were analyzed: 1) One patient with a PFO with a large left atrial opening (4.5 mm) and a long tunnel (13.5 mm) was implanted with a 30/30 mm occluder. One year later, the left umbrella disc was degraded to 8 mm and did not completely cover the left atrial opening of the PFO. A tunnel-like channel was formed in the occluder (Figure 7). 2) One patient with a PFO with a small left atrial opening (1.5 mm) and a short tunnel (3 mm) was implanted with a 24/24 mm occluder. The longer waist (4 mm) of the occluder caused a poor fit of the device disc and the atrial septum to form a small tunnel in the occluder. Multiple studies have demonstrated the presence of a residual PFO shunt in 2–10% of patients at the 6-month follow-up after percutaneous PFO closure. The majority of residual shunts occur around the existing device; however, a subset of patients exhibit an intradisc shunt (32, 33), as were the cases with our patients. The previous literature reports shown, transpulmonary passage of small and moderate contrast bubbles may be traveling through larger-diameter vessels. There is direct evidence that these larger-diameter (>25–50μm) intrapulmonary arteriovenous anastomoses exist in healthy human, baboon, and dog lungs (34). Furthermore, Stickland et al. have directly demonstrated that these pathways are dormant at rest in healthy dogs but open up during exercise. The intrapulmonary arteriovenous vessels that allow for the transpulmonary passage of saline contrast bubbles during normoxic and hypoxic exercise in adult humans may be remnant fetal pathways (35). In our study, 1 year after occlusion, the small shunts detected at the level of the pulmonary veins by TEE may be originating from these physiological intrapulmonary arteriovenous anastomoses.

The current study has some limitations. To simplify the operation procedure, the biggest limitation of this study was that all patients underwent PFO occlusion with NBO under TTE rather than TEE guidance. This meant that the detailed anatomical relationship between the occluder and PFO and the atrial interval were not obtained during the operation. Moreover, because of the small number of patients, the true incidence of residual shunts and thrombi remains unknown. Finally, the follow-up time was short, and the long-term effects need to be further investigated.

## Conclusion

In the present study, we demonstrated the feasibility, safety, and efficacy of a NBO for the treatment of PFO in humans. PFO closure was achieved in 95.5% of the patients. NBO provides high long-term closure rates and safety. In this procedure, TEE has a major role in the diagnostic and anatomical evaluation of PFO, as well as in diagnosing potential complications or outcomes (such as device thrombosis, device size change, and cardiac or extracardiac residual RLS) on Post-procedure follow-up. In particular, TEE has unique advantage in detecting the specific reasons for an extensive residual shunt in the late postoperative period.

## Data Availability Statement

The raw data supporting the conclusions of this article will be made available by the authors, without undue reservation.

## Ethics Statement

The studies involving human participants were reviewed and approved by Ethics Committee of the First Affiliated Hospital of Xi'an Jiaotong University (No: XJTU1AF2015LSL-049). The patients/participants provided their written informed consent to participate in this study.

## Author Contributions

YD and YZ contributed to study concept and design. HX, LH, and GC contributed to data analysis and interpretation and critical revision of the article. YD and HX contributed to drafting of the article. HS, XW, XH, and BL read and approved the final manuscript. All authors contributed to the article and approved the submitted version.

## Funding

This study was supported by Shaanxi Province Key Industry Innovation Chain (Group)-Social Development Field (No.2020ZDLSF04-14).

## Conflict of Interest

The authors declare that the research was conducted in the absence of any commercial or financial relationships that could be construed as a potential conflict of interest.

## Publisher's Note

All claims expressed in this article are solely those of the authors and do not necessarily represent those of their affiliated organizations, or those of the publisher, the editors and the reviewers. Any product that may be evaluated in this article, or claim that may be made by its manufacturer, is not guaranteed or endorsed by the publisher.
